# How Online Scheduling Platforms Affect Insurance-Based Disparities in Access to Specialist Outpatient Care in Berlin, Germany: Cross-Sectional Audit Study

**DOI:** 10.2196/82452

**Published:** 2026-06-15

**Authors:** Cordula Charlotte Josefine Kreuzenbeck, Silvia Angerer

**Affiliations:** 1 IU International University of Applied Science, Juri-Gagarin-Ring 152, Erfurt, 99084, Germany, 49 1794425752; 2 Department for Public Health, Health Services Research and Health Technology Assessment, UMIT TIROL—Private University for Health Sciences and Health Technology, Hall in Tirol, Austria

**Keywords:** health services accessibility, health care disparities, insurance, algorithms, medical informatics applications, internet, health policy, health

## Abstract

**Background:**

Online scheduling platforms are increasingly chosen by patients for scheduling outpatient appointments. Due to payment for listing or platform decisions on listing visibility, they can amplify access inequalities. Especially in Germany’s dual insurance system, the beneficiary difference in waiting times for private health insurance (PHI) patients compared to statutory health insurance (SHI) patients for specialist appointments might increase.

**Objective:**

The objective of this study was to quantify differences in waiting times for the first available specialist outpatient appointments for SHI versus PHI profiles on a large commercial online scheduling platform (Doctolib) in Berlin and to assess whether practices offering equal appointment options for both insurance types are deprioritized by the platform.

**Methods:**

We performed a cross-sectional, internet-based audit between January 6, 2025, and February 26, 2025. Two standardized simulated patient profiles (SHI and PHI) were used to query 1867 platform listings; practices with at least 1 bookable slot for both profiles were documented (n=492). The primary outcome was the waiting time in days for the earliest available appointment. Paired 1-tailed *t* tests and Wilcoxon signed-rank tests were used to compare within-provider waiting times, while exploratory subgroup analyses examined specialty-specific differences. Sensitivity analyses excluded the top 5% of the longest waits. Effect sizes (Hedges *g*) were computed.

**Results:**

Across 492 practices, mean waiting times were 50.4 (SD 52.2) days for SHI and 22.6 (SD 36.8) days for PHI, yielding a mean difference of 27.8 days (95% CI 23.6‐31.9 d; *t*_491_=13.05; *P*<.001; Hedges *g*=0.59). After excluding the top 5% of waiting times (n=44), the difference remained significant (Δ=23.1 d; 95% CI 20.0‐26.2 d; *t*_447_=14.77; *P*<.001). Providers that offered earlier PHI appointments showed substantially longer SHI waiting times (61.8 vs 37.5 d; *P*<.001) and substantially shorter PHI waits (12.0 vs 38.2 d; *P*<.001). Platform ranking was correlated with earlier PHI availability (Spearman rank correlation *r*=0.574; *P*<.001).

**Conclusions:**

On this commercial online scheduling platform in Berlin, SHI patients experienced substantially longer waits for the first available specialist appointments than PHI patients across multiple specialties. Potential mechanisms that could amplify pre-existing insurance-based access inequities are discussed. Transparency about the platform’s ranking criteria should be considered to promote equitable access.

## Introduction

Disparities in access to medical care—particularly in scheduling outpatient appointments—are well-documented in health services research [1-3]. In Germany, this issue is especially pronounced due to its dual insurance system, which separates statutory health insurance (SHI) and private health insurance (PHI). Approximately 11% of the population is insured under PHI [4].

Field experiments have shown that SHI patients face significantly longer waiting times than PHI patients when attempting to schedule specialist appointments by phone, with reported differences ranging from 7 to 15 days [5-9]. Earlier data found that, in 2006, average waiting times for specialist appointments ranged from 9.5 to 24.8 working days [10]. In contrast, waiting time disparities have not been observed to the same extent in the primary care sector in Germany [11].

Recently, online scheduling platforms have become increasingly common and may soon replace traditional phone-based scheduling in many settings [12,13]. Atherton et al [12] report a mixed methods investigation of patient use and experience with online scheduling platforms in primary care, focusing on adoption, usability, and user perceptions rather than audit outcomes [12]. Studies that examine online scheduling platforms find lower no-show rates and overall shorter waiting times but do not assess inequalities [13,14].

While online scheduling platforms offer convenience and the appearance of neutrality, emerging evidence suggests they may also perpetuate or even reinforce access inequities. A US-based study of an online scheduling platform in primary care found systematic discrimination against Medicaid patients. Kurtzman et al conducted an audit of online primary care appointment availability in the United States and identified differences in availability by insurance status (Medicaid vs private insurance). Although over 4000 listings were analyzed, this study did not simulate real bookings and did not focus on specialist care [15]. Our study adds a cross-specialty, Germany-based audit on a commercial online scheduling platform, extending the online-audit literature from primary care settings in the United States to specialist bookings in a European dual insurance system.

Online scheduling platforms can implement algorithms and rules that may be discriminatory by design, which can be enforced through the application of machine learning [16]. Particularly with the power to decide how the algorithm determines visibility on the listing page and the position in the list, online scheduling platforms can influence the metrics regarding how practices offer appointment slots to patients.

This study adds field evidence that disparities based on insurance status are present on a commercial online platform in practice. The analyzed platform, Doctolib, is a self-reported leading online scheduling platform in Europe. It introduces an active process of applying rules to the platform, through which patients can access appointments, and it comes with a monthly fee for physicians.

This study aims to investigate whether SHI patients experience longer waiting times than PHI patients when booking specialist appointments through an online scheduling platform. Specifically, we examine (1) the magnitude of waiting time differences between SHI and PHI patients across multiple medical specialties on the platform and (2) whether practices offering equal appointment options for both insurance types are deprioritized by platform algorithms. In doing so, our study contributes to the growing literature on digital health access by extending online audit methodologies beyond primary care and the US context. It provides the first cross-specialty, Germany-based audit of a commercial online scheduling platform, offering empirical evidence that platform design may potentially reinforce existing disparities in access to specialist care in a dual insurance system.

## Methods

### Study Design

We conducted a cross-sectional, internet-based audit study to evaluate differences in appointment availability for SHI versus PHI patients on Doctolib in Berlin, Germany. The study follows the STROBE (Strengthening the Reporting of Observational Studies in Epidemiology) guidelines (Checklist 1) for cross-sectional research and adopts a “simulated patient” methodology to minimize measurement bias [17].

### Setting and Data Source

Data were collected between January 6, 2025, and February 26, 2025. The platform aggregates publicly accessible listings for outpatient medical specialists in Berlin and allows users to filter by insurance status (SHI vs PHI). To mitigate temporal variability, each specialty was assessed within 1 day for both the PHI and the SHI profiles. Due to frequent platform updates, the order of appearance changed throughout the assessment window. To ensure complete uptake of all available specialists, all search result pages were saved as static HTML snapshots at the moment of extraction. These snapshots were then double-checked to confirm that all possible appointments were incorporated. For validation, the assessment day was included as a possible confounder.

### Participants and Sample Selection

The specialties were derived from the European Union Professional Qualifications Directive (2005/36/EC) of the European Parliament and of the Council of September 7, 2005, on the recognition of professional qualifications [18]. The study excluded the specialties of dentistry and orthodontics, which showed no observable insurance-based differences in a prior separate prestudy conducted in Frankfurt. Additionally, for these specialties, there was no option to select between SHI and PHI provided on Doctolib. We screened 1867 provider listings on the platform; inclusion criteria were as follows: (1) active online booking availability during the data collection window and (2) availability of appointments for both SHI and PHI. Providers offering only 1 insurance type or no online booking were excluded. A total of 492 practices met the inclusion criteria and were analyzed.

### Audit Simulation and Data Collection Procedure

The following provides a step-by-step overview of the data collection procedure. First, 2 standardized profiles were created: profile 1 representing an SHI patient and profile 2 representing a PHI patient (profile 1: male, aged 42 y, no prior appointments or medical history on the platform; profile 2: female, aged 36 y, no prior appointments or medical history on the platform).

Next, the online scheduling platform Doctolib page was opened, and the specialty was chosen. According to the profile, the filter for PHI or not was chosen. For each specialty, 2 parallel searches—1 under each profile—were conducted on the same day and, wherever possible, within the same browser session to control for diurnal fluctuations in availability. For multiphysician practices or multilocation listings, each distinguishable physician or location combination was treated as a unit. For each unit (listing of a physician), the earliest available appointment date without completing a booking by following the booking process was recorded. For simplicity, we refer to these units as “practices” throughout the paper, even though a single practice may have multiple listings.

To avoid systematic variation in the type of appointment requested, 1 of 2 standard booking options, identical for both patient profiles, was consistently used: (1) at priority 1, “first consultation new patient,” and if this was not available, (2) “unspecific appointment within consultation hours” was chosen for all specialties, reflecting a typical new patient inquiry. The only exception was radiology, where we consistently selected magnetic resonance tomography or computed tomography (hand; magnetic resonance tomography priority 1 and computed tomography priority 2) because no general appointment categories were offered.

Before the final booking, the day available for booking was noted, and the process was stopped. No real appointments were made, ensuring that study activities did not impact providers’ schedules. Each page was saved as a screenshot before moving on to the next page of listings. Doctolib provides 20 listings per page. All listings were documented (name of listing or practice and, in 3 specialties, also the address), including those that did not offer any appointments for any profile, which led to the final dataset with practices providing appointments for both PHI and SHI.

The process was repeated for each practice and profile until the last provider within Berlin. The first practice outside of Berlin was identified by the platform, which provided a note on the kilometer distance to Berlin and the name of the other city. When 1 specialty was fully assessed by both profiles and validation was done, the process was repeated for a different specialty on the same or another day.

After data collection for both profiles in each specialty, the insurance status was switched between the 2 profiles (female to SHI and male to PHI), and the first 20 listings were reassessed to investigate potential confounding with gender- or age-based discrimination due to profile construction.

The full list of variables extracted from Doctolib can be accessed in Multimedia Appendix 1. For the first research question on waiting time disparities, we used waiting times in days for the SHI and PHI profiles on an appointment for each practice as the primary outcome. Waiting times were defined as the difference in days between the day of the “First available appointment” and the “Day of data assessment.” For subgroup analysis, we differentiated between the specialties of practice listings. The study also included the “Rank on Doctolib” for some specialties to address the second research question.

### Bias Control

To reduce measurement bias and maintain consistency, the data collection was conducted in the order presented by the platform (first to last page and top to bottom), and static snapshots were archived.

### Statistical Analysis

Descriptive statistics (mean and median) were computed for waiting times by insurance type. Paired 1-tailed *t* tests and nonparametric Wilcoxon signed-rank tests were used to assess within-provider differences in waiting times for SHI versus PHI. To explore heterogeneity, subgroup analyses stratified by specialty were conducted; however, given the small sample sizes, the results are descriptive only and should be interpreted cautiously. All analyses were 1-tailed with α=.05. To adjust for multiple comparisons when stratifying by specialty, a conservative Bonferroni correction was applied, which involves multiplying the unadjusted *P* values by the number of hypotheses (in our case, subgroups) tested [19]. Analyses were performed in SPSS, version 28 (IBM Corporation). Effect sizes (Hedges *g*) were computed.

### Sensitivity Analysis

To evaluate the robustness of the findings, we excluded the top 5% of practices with the longest waiting times (outliers) and repeated the paired comparisons. This outlier exclusion did not materially alter the direction or significance of the overall results; however, some specialty subgroups lost sufficient power for significance.

### Ethical Considerations

This study involved only publicly available booking information and did not involve any real patient data, physician data, or actual appointment bookings. Neither were provider schedules impacted, nor were provider names reported. Due to the study design being observational and not involving any human participants, no formal ethics approval was applied for. This follows the guidelines from the German Research Society, where none of the cases that require an ethical vote apply to this study design [20].

## Results

### Listings and Bookable Practices

**Figure 1.**
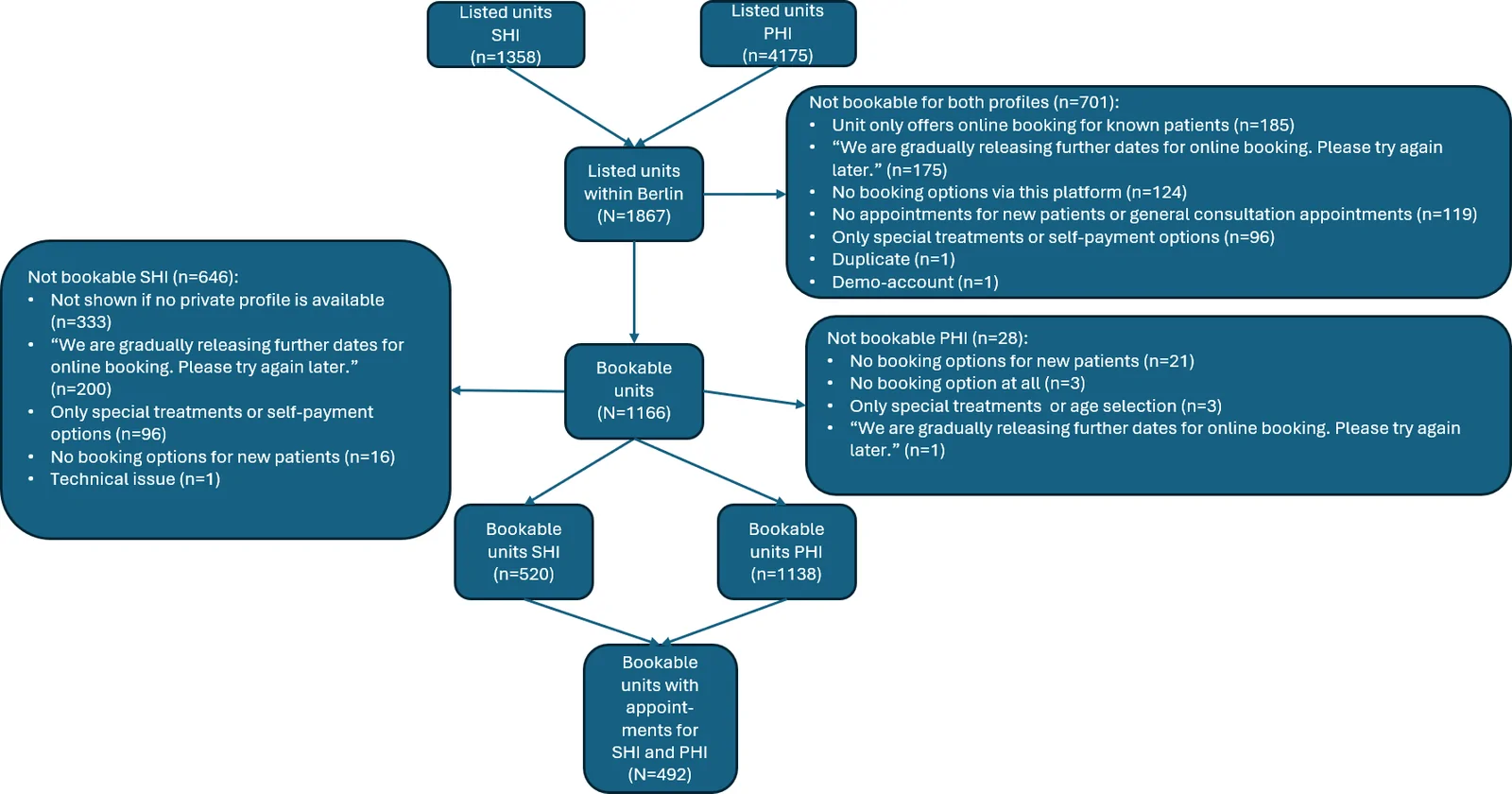
Extraction of listings, exclusions with exclusion criteria, and final dataset. Units: listings of practices. PHI: private health insurance; SHI: statutory health insurance.

A total of 1867 provider listings were screened on the platform between January 6, 2025, and February 26, 2025. After applying our exclusion criteria (no online booking, availability for only 1 insurance type, or specialty outside the predefined list), 492 practices remained with at least 1 bookable appointment slot for both SHI and PHI patients (bookable practices with appointments for both groups). Figure 1 illustrates the detailed inclusion and exclusion processes.

Multimedia Appendix 2 compares bookable practices with all available practices in Berlin using data from the Association of Statutory Health Insurance Physicians (Kassenärztliche Vereinigung [KV]) to estimate the platform’s approximate local market share. Note that the Berlin KV registry lists all outpatient specialists authorized to treat statutory-insured patients; however, platform listings include multiple entries per physician and might omit some practices, so the KV comparison is approximate.

Overall, 492 of 4123 physicians listed on the platform offer services to SHI patients, accounting for approximately 12.6% of the available practices in the relevant specialties in Berlin, according to the KV. This estimate should be considered a lower bound, as the lists do not correspond perfectly. In particular, the KV categories comprise broader specialty groupings (eg, neurology or psychiatry) than those represented on Doctolib, which leads to discrepancies in the comparison.

### Provider Bookability by Specialty

Table 1 provides an overview of the distribution of cases among the specialties, the day of observation, and the descriptive results on waiting times.

The specialties with the highest SHI bookability rates were ENT (ear, nose, and throat), orthopedics, obstetrics and gynecology, and urology. Neurology had the lowest SHI bookability rate (0.5%), with only 2 out of 405 registered neurologists offering SHI appointments on this platform online.

**Table 1.** Descriptive statistics of waiting times for statutory health insurance (SHI) and private health insurance (PHI) patients by practice specialization.

Specialty	n	Day of observation	PHI<SHI	No difference	SHI<PHI	PHI<SHI (%)	SHI, mean (SD); median (IQR)	PHI, mean (SD); median (IQR)
General surgery	13	Tuesday	7	5	1	53.8	51.9 (40.0); 57 (21-69)	17.0 (21.9); 8 (2-21)
Ophthalmology	20	Sunday	8	11	1	40	48.6 (58.3); 19 (2-86)	17.2 (35.0); 3 (2-9)
Obstetrics and gynecology	103	Sunday	46	41	16	44.7	57.9 (54.7); 44 (15-86)	37.9 (48.6); 19 (4-58)
Vascular surgery	3	Sunday	0	2	1	0	39.7 (39.5); 38 (1-80)	51.7 (24.5); 38 (37-80)
Dermatology	18	Monday	6	11	1	33.3	63.0 (43.7); 55 (28-99)	47.4 (44.1); 36 (9-78)
ENT^a^	75	Wednesday	45	27	3	60	50.6 (35.9); 49 (21-78)	19.9 (29.5); 6 (1-23)
Cardiology	14	Monday	11	2	1	78.6	174.8 (123.2); 206 (52-283)	44.1 (95.8); 2 (0-23)
Pediatrics	12	Sunday	0	12	0	0.0	13.5 (18.6); 2 (1-26)	13.5 (18.6); 2 (1-26)
Neurology	2	Monday	1	1	0	50.0	82.0 (5.7); 82 (78-86)	81.5 (6.4); 82 (77-86)
Orthopedics	159	Tuesday	121	29	9	76.1	36.2 (31.4); 29 (13-55)	11.2 (16.8); 6 (1-13)
Radiology	38	Sunday	15	21	2	39.5	19.6 (25.2); 7 (2-25)	8.9 (16.8); 2 (1-8)
Urology	35	Saturday^b^	23	10	2	65.7	81.7 (52.5); 89 (47-107)	31.6 (32.2); 20 (4-58)
All specialties	492	—^c^	283	172	37	57.5	50.4 (52.2); 37 (10-74)	22.6 (36.8); 7 (1-29)

a ENT: ear, nose, and throat.

b Because the day following the observation was a Sunday—on which no bookings could be made—waiting times for both SHI and PHI patients were systematically reduced by 1 day.

c Not applicable.

Paired *t* tests and nonparametric Wilcoxon signed-rank tests were conducted across specialties to compare the first available appointment waiting times for SHI versus PHI profiles. Specialties with fewer than 3 evaluable cases (vascular surgery and neurology) were excluded from hypothesis testing. Pediatrics did not show any differences, so hypothesis testing could not be performed. Table 2 summarizes the results.

Across all specialties (including those with small sample sizes), SHI patients waited, on average, 27.8 (47.2) days longer than PHI patients (95% CI 23.6‐31.9; *t*_491_=13.05; *P*<.001; Hedges *g*=0.59), corresponding to approximately 4 weeks.

At the specialty level, statistically significant differences in waiting times between SHI and PHI patients were observed in 8 out of 9 specialties (*P*<.05). After applying a Bonferroni correction to account for multiple comparisons, 6 of these differences remained statistically significant (denoted as footnote “a” in Table 2), indicating that the overall pattern is not solely driven by chance findings. The largest disparities occurred in cardiology and urology. Results at the specialty level should, however, be interpreted cautiously, given the small sample sizes.

Given the skewed distribution of waiting times in some specialties, nonparametric Wilcoxon signed-rank tests are reported alongside *t* tests. The results remain qualitatively unchanged.

**Table 2.** Paired *t* test and Wilcoxon signed-rank test results for the differences between statutory health insurance (SHI) and private health insurance (PHI) waiting times for the first available appointment by practice specialization.

Specialty^a^	n^b^	SHI–PHI, mean difference^c^ (SD)	95% CI	*t* test^d^		Wilcoxon signed-rank test^e^, *P* value	Hedges *g*^f^
				*t* test (*df*)	*P* value		
General surgery	13	34.9 (42.7)	9.12 to 60.72	2.95 (12)	.01	.02	0.79
Ophthalmology	20	31.4 (46.5)	9.65 to 53.16	3.02 (19)	.01	.01	0.66
Obstetrics & gynecology	103	20.1 (43.7)	11.51 to 28.61	4.65 (102)	<.001	<.001	0.46
Dermatology	18	15.6 (31.6)	–0.10 to 31.33	2.10 (17)	.05	.05	—^g^
ENT^h^	75	30.7 (40.4)	21.43 to 40.02	6.59 (74)	<.001	<.001	0.76
Cardiology	14	130.7 (125.8)	58.11 to 203.32	3.89 (13)	.002	.003	1.01
Pediatrics	12	0	—	—	—	—	—
Orthopedics	159	25.0 (31.7)	20.00 to 29.93	9.93 (158)	<.001	<.001	0.79
Radiology	38	10.7 (22.5)	3.28 to 18.09	2.92 (37)	.01	.001	0.47
Urology	35	50.0 (56.6)	30.57 to 69.48	5.23 (34)	<.001	<.001	0.87
All specialties	492	27.8 (47.2)	23.57 to 31.93	13.05 (491)	<.001	<.001	0.59

a Specialties in obstetrics and gynecology, ENT, cardiology, orthopedics, radiology, and urology indicate statistically significant differences (*P*<.05) after Bonferroni correction.

b Reports the number of observations.

c Presents the mean difference in waiting times (in days) between the first available appointment for SHI and PHI patients.

d Report the results of the 1-tailed *t* test.

e Report the results of the Wilcoxon signed-rank test.

f Provides the effect size, calculated as Hedges *g*.

g Not available.

h ENT: ear, nose, and throat.

**Figure 2.**
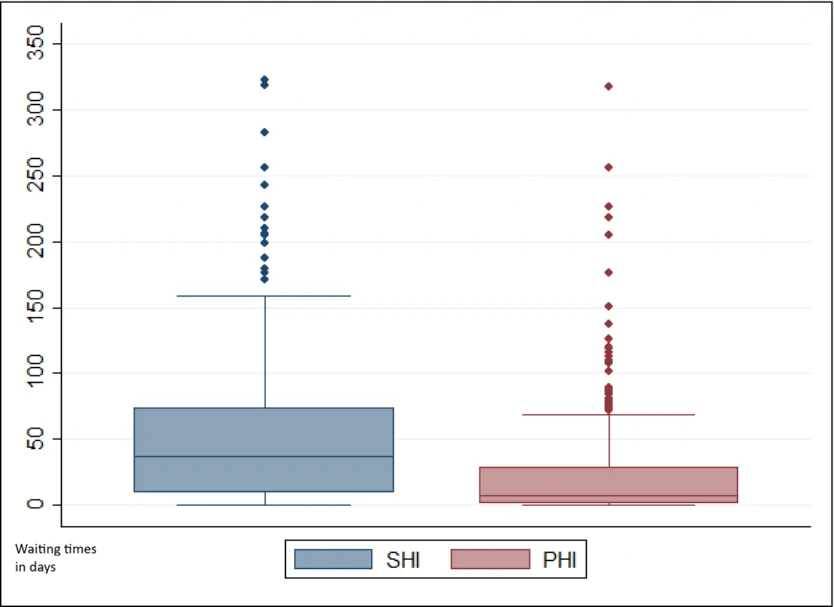
Boxplot showing the distribution of waiting times for the first available appointment, comparing statutory health insurance (SHI) and private health insurance (PHI) insurance profiles.

Figure 2 provides a boxplot showing the statistically significant difference in waiting times across all specialties between the SHI and PHI profiles.

### Comparison of Providers With and Without Faster PHI Appointments

Given that physicians differentiate appointment allocations between SHI and PHI patients, we investigated whether longer waiting times for SHI patients correspond to shorter waiting times for PHI patients. Therefore, we examined whether offering faster appointment slots to PHI patients was associated with waiting times for both SHI and PHI profiles. Providers were classified into 2 groups as follows:

- Group 0 (practices with equal SHI and PHI appointment timing): providers whose earliest SHI and PHI appointments did not differ (n=172).
- Group 1 (practices with earlier PHI than SHI availability): providers whose PHI appointment was earlier than their SHI appointment (n=283).

A total of 37 practices that offered an earlier appointment to SHI patients were excluded from this analysis.

Table 3 shows that SHI waiting times were significantly longer for group 1 (61.9 d) compared with group 0 (37.6 d), with a mean difference of −24.3 days (95% CI −33.6 to −14.9 d; *t*_454_=−5.10, *P*<.001; Hedges *g*=−0.469). SHI patients wait 24.3 days less when they visit practices from group 0.

PHI waiting times were significantly shorter in group 1 (12.1 d) compared with group 0 (37.6 d), with a mean difference of 25.5 days (95% CI 18.2‐32.9 d; *t_454_*=6.81, *P*<.001; Hedges *g*=0.75). This shows that the group of practices with earlier PHI availability than SHI availability can offer appointments to PHI patients 25.5 days earlier than those that do not differentiate between SHI and PHI patients.

These results indicate that practices offering faster slots to PHI patients not only reduced PHI waiting times substantially but also correspondingly increased SHI waiting times, with large effect sizes in both comparisons.

For the specialties of obstetrics and gynecology, dermatology, urology, and ENT (n=801), we were able to reproduce the ranking on Doctolib exactly due to the exact name and address notation in our dataset and the corresponding screenshots from the search with the PHI profile. With 493 practices having available rankings and PHI appointments, we performed the following correlation analysis: rank on Doctolib (place of listing where 1 is the first listing on the page and counting) and waiting time for PHI have a highly significant (*P*<.001) Spearman rank correlation *r* of 0.702. This indicates a strong positive correlation. These findings demonstrate that the ranking order of entries on Doctolib is associated with appointment availability. Specifically, listings offering the earliest available appointments are positioned highest in the ranking.

Of the specialties with information on the rank, 175 also had appointments available for both PHI and SHI patients. An unpaired *t* test showed that practices in group 0 are, on average, 58 ranks behind compared to group 1 (95% CI 37.9-78.0; *t_174_*=5.706, *P*<.001; Hedges *g*=0.86). As the listings on Doctolib have 20 practices per page, practices with equal SHI and PHI appointment timing will, on average, be placed almost 3 pages behind practices with earlier PHI than SHI availability on this platform in the view of PHI profiles.

**Table 3.** Descriptive statistics, tests, and effect sizes for waiting times by provider group^a^.

Variable and group	n	Mean (SD)	Group 0–group 1, mean difference (95% CI)	1-tailed *t* test		Levene *F* test		Effect sizes: Hedges *g*^b^
				*t* test (*df*)	*P* value	*F* test (df)	*P* value	
Wait time (SHI)^c^			−24.3 (−33.60 to −14.91)	−5.104 (454)	<.001	4.208 (454)	.04	−0.469
Group 0	172	37.6 (45.1)						
Group 1	283	61.9 (55.2)						
Wait time (PHI)^d^			25.5 (18.15 to 32.92)	6.810 (454)	<.001	61.094 (454)	<.001	0.747
Group 0	172	37.6 (45.1)						
Group 1	283	12.1 (25.3)						

a Levene test assessed equality of variances. Mean difference (0–1): group 0 (practices with equal SHI and PHI appointment timing) mean minus group 1 (practices with earlier PHI than SHI availability) mean (so negative for SHI indicates longer waits in group 1).

b Effect sizes: Hedges *g* used pooled SD.

c SHI: statutory health insurance.

d PHI: private health insurance.

### Validation Analysis: Exclusion of the Top 5% of Outliers

To confirm the robustness of our primary findings, we repeated the paired *t* tests and Wilcoxon signed-rank tests after excluding the top 5% (n=44) of the longest waiting times, yielding a sample of 448 practices. Table 4 presents the group statistics and inferential results for SHI versus PHI waiting times in this reduced sample by specialists and overall.

After removing extreme waiting time outliers in both SHI and PHI patients, SHI patients continued to experience significantly longer waits than PHI patients, with an average difference of 23.1 days (95% CI 20.0‐26.2; *t*_447_=14.77; *P*<.001). This validation analysis confirms the primary finding of a substantial disparity in appointment waiting times based on insurance status.

At the specialty level, statistically significant differences between SHI and PHI waiting times were observed in 7 of 12 specialties; 5 of these remained significant after Bonferroni correction (denoted as footnote “a” in Table 4). The largest disparities were found in the specialties of urology, general surgery, and ENT.

**Table 4.** Paired *t* test and Wilcoxon signed-rank test results for the difference between statutory health insurance (SHI) and private health insurance (PHI) waiting times for the first available appointment by practice specialization after excluding the top 5% of outliers.

Specialty^a^	n^b^	SHI–PHI, mean difference^c^ (SD)	95% CI	*t* test^d^ (df)		Wilcoxon signed-rank test^e^, *P* value	Hedges *g*^f^
				*t*	*P* value		
General surgery	13	34.9 (42.7)	9.1 to 60.7	2.949 (12)	.01	.02	0.766
Ophthalmology	16	22.4 (33.0)	4.8 to 39.9	2.714 (15)	.02	.04	0.644
Obstetrics and gynecology	89	17.4 (32.3)	10.6 to 24.2	5.075 (88)	<.001	<.001	0.533
Vascular surgery	3	−12.0 (20.8)	—^g^	—	—	—	—
Dermatology	13	15.2 (25.9)	−0.4 to 30.9	2.119 (12)	.06	.02	0.550
ENT^h^	71	31.6 (36.8)	22.9 to 40.3	7.235 (70)	<.001	<.001	0.849
Cardiology	5	24.8 (34.0)	−17.4 to 67.0	1.630 (4)	.18	.17	0.582
Pediatrics	12	—	—	—	—	—	—
Neurology	1	—	—	—	—	—	—
Orthopedics	156	23.3 (27.7)	19.0 to 27.7	10.530 (155)	<.001	<.001	0.839
Radiology	38	10.7 (22.5)	3.3 to 18.1	2.924 (37)	.01	<.001	0.465
Urology	31	45.8 (48.7)	27.9 to 63.6	5.234 (30)	<.001	<.001	0.916
All specialties	448	23.1 (33.1)	20.0 to 26.2	14.77 (447)	<.001	<.001	0.59

a Specialties in obstetrics and gynecology, ENT, orthopedics, radiology, and urology indicate statistically significant differences (*P*<.05) after Bonferroni correction.

b Reports the number of observations.

c Presents the mean difference in waiting times (in days) between the first available appointment for SHI and PHI patients.

d Report the results of 1-tailed *t* test.

e Report the results of the Wilcoxon signed-rank test.

f Provides the effect size, calculated as Hedges *g*.

g Not available.

h ENT: ear, nose, and throat.

As with the main specialty-level analyses, these findings should be interpreted cautiously, given the small sample sizes within individual specialties, which may affect the precision and stability of the estimates.

### Validation Analysis: Profile Construction and Potential Confounding

To assess potential confounding due to gender- and age-based discrimination in profile construction, we reassessed the first 20 listings within each specialty after switching the insurance status assigned to the 2 profiles. Across all specialties, the earliest available appointment date remained consistently linked to the insurance status indicated in the profile rather than to the gender or age of the profile. Specifically, when the male profile, aged 42 years, was assigned PHI status, it received the same earliest appointment option previously observed for the female PHI profile, aged 36 years, and vice versa for SHI. No deviations from this pattern were observed. These findings suggest that the differences in waiting times are most likely attributable to insurance status rather than gender- or age-related factors embedded in the profile design.

## Discussion

### Main Study Findings

In this cross-sectional, internet-based audit of 492 outpatient specialists listed on a major European booking platform, we found that SHI patients in Germany faced substantially longer waiting times for the first available appointments compared with PHI patients. To our knowledge, no previous study of comparable scale has examined appointment allocation via online scheduling platforms in a dual insurance system of PHI and SHI, specifically for specialist care.

Across multiple medical specialties, SHI patients waited, on average, 23 to 32 days longer than PHI patients. Additionally, for PHI patients, there were more than twice as many practices with offerings listed on the platform compared to SHI patients in the same region.

Although more than one-third of practices offered identical appointment dates to both profiles, these physicians appeared at a significantly later rank in search results and had significantly longer waiting times for PHI patients. The practices with earlier PHI than SHI availability, on the other hand, showed significantly longer waiting times for SHI patients and had a higher rank in the listing. Thus, the preferential ranking of earlier appointment slots advantages practices whose PHI slots precede their SHI slots.

### Analysis of the Findings

Our findings indicate that SHI patients face significant disparities in outcomes on the platform, both in terms of reduced appointment availability and longer waiting times. The observed difference in waiting times, averaging 23 to 32 days, is larger than what was demonstrated in earlier telephone-based audit studies in Germany [5-10]. For example, Breitenbach and Heinrich [5] documented waiting times for SHI patients that were, on average, 15 days longer than for PHI patients in major German cities. Similarly, Heinrich et al [6] reported differences of 14 to 23 days for elective procedures in a high-density urban region (excluding Berlin). Other studies found smaller gaps: in Bavaria, Muschol and Gissel [8] observed differences of 9 days in 2019 and 6 days in 2020, while Werbeck et al [9] reported an average difference of 13 weekdays across 36 counties in all 16 German federal states. Lungen et al [10] found differences ranging from 5 to 25 working days depending on the specialty and the requested intervention. This variation across specialties mirrors the pattern observed in our study, where disparities between SHI and PHI patients also differ substantially by medical specialty. By contrast, disparities in primary care appear much smaller; Luque Ramos et al reported differences of less than 1 day [11].

Taken together, these comparisons suggest that disparities observed through online scheduling platforms are at the upper end—or exceed—the range documented in prior German studies using telephone-based appointment scheduling. Evidence on insurance-based differences in appointment allocation on online scheduling platforms is scarce. The only study investigating insurance-based differences on online scheduling platforms was conducted in the United States and examined appointment scheduling with primary care physicians [12]. It found no differences in the timing of available appointments by insurance status; however, patients insured through Medicaid were directed to physicians located farther away, indicating disparities in geographic access. Comparability with this study is, however, limited, as the analysis was conducted within the US health care system and focused on primary care, whereas this study examines access to outpatient specialist appointments in the German context of statutory and private health insurance.

Turning to the mechanism that may help explain the observed disparities in appointment availability and waiting times: one explanation relates to economic incentives within the dual insurance system. Reimbursement levels for PHI patients are generally higher and less tightly regulated than those for SHI patients, potentially incentivizing practices to allocate scarce appointment slots preferentially to PHI patients. Consistent with this interpretation, our audit revealed that a notable subset of practices (n=96) presented additional self-pay or privately billed service options exclusively to SHI profiles. These offers often took the form of enhanced screening packages, extended consultation times, self-pay appointments, or cosmetic procedures, and were either absent or framed differently for the PHI profile. This pattern suggests that some practices actively promote out-of-pocket services to SHI patients as an alternative revenue stream. Such differential presentation of self-pay options not only adds another layer of complexity to access—where SHI patients face not only longer waiting times but also additional financial considerations—but may also reflect incentives to prioritize higher-margin services over standard insured care. From a health-economics perspective, these dynamics could exacerbate inequities by shifting SHI patients toward more costly, noncovered services.

Building on this, and in connection with our result that practices with earlier PHI than SHI availability are ranked higher in the platform listing, the platform’s ranking logic—while not explicitly designed to favor PHI patients—could indirectly amplify differences in access by directing patients toward practices where PHI patients receive faster appointments. In this sense, ranking based on waiting times may introduce a second layer of prioritization: practices that already allocate appointments preferentially to PHI patients become more visible and attractive within the platform environment. This dynamic may create reinforcing incentives for practices to prioritize PHI patients, since shorter waiting times improve their relative ranking and potentially attract additional demand. Over time, such feedback mechanisms could widen inequalities in outpatient care access between SHI and PHI patients, even if the platform’s design does not explicitly intend to differentiate by insurance status.

While differential reimbursement and platform ranking are potential mechanisms, the observed pattern may also reflect alternative explanations, such as differences in expected case complexity, expected consultation length, or differential demand patterns between SHI and PHI patients. Because the audit design cannot distinguish empirically between these alternative explanations, further research should investigate the underlying mechanisms and whether, and to what extent, ranking amplifies insurance-based disparities in access to specialist care.

Although the share of practices using Doctolib is still limited in Berlin, the use of online scheduling platforms is on the rise [12,13], and other platforms work in a similar manner (eg, Jameda). Thus, although this study was conducted in Berlin using Doctolib, the results are likely informative for a broader population of patients and providers actively using online scheduling platforms in other regions and on other platforms. Further research is needed to assess the generalizability of these findings across different regions and online scheduling platforms.

These findings also raise questions about the role of platform design in shaping access to care. From a policy perspective, this suggests that safeguards—such as algorithmic audits or insurance-neutral ranking criteria—could be considered to mitigate the risk of unequal access. Greater transparency regarding the factors that influence platform rankings may also be important to better understand how digital scheduling systems affect the distribution of appointments across patient groups.

### Limitations

This study offers a valuable contribution by systematically quantifying insurance-based disparities in access to specialist care through online scheduling platforms, a growing channel in health care delivery. Nevertheless, several limitations warrant consideration.

First, our sampling frame comprised only practices listed on a single online platform; approximately 12.6% of KV‐registered specialists offered SHI appointments on this platform. Our results, therefore, do not capture the full range of outpatient care pathways, including appointment scheduling via phone or in‐person. Additionally, the sample is not representative as the opt-in to the paid use of this platform is likely to induce a selection bias by itself. Providers participating in online scheduling platforms are likely a nonrandom subset of all outpatient specialists. Adoption may be higher among digitally oriented practices, larger group practices, or those serving a higher share of PHI or self-paying patients. In addition, practices facing higher demand or capacity constraints may have stronger incentives to actively manage appointment allocation via digital tools. As a result, our findings are not generalizable to the appointment scheduling behavior of all outpatient physicians. This selection into the platform could affect the magnitude of the observed disparities. On the one hand, practices adopting digital scheduling tools may have more standardized appointment management and greater transparency in slot allocation, which could attenuate differences between SHI and PHI patients compared with traditional scheduling channels. On the other hand, digital platforms may facilitate more explicit segmentation of appointment slots or patient groups, potentially amplifying differences in waiting times if practices strategically allocate capacity. Compared with evidence from telephone-based appointment scheduling studies, the latter explanation seems more plausible [5,6,8-11]. Future research should, therefore, explicitly examine the determinants of providers’ participation in online scheduling platforms and how such selection may influence observed patterns of access.

Nevertheless, even if our findings are not generalizable to the appointment scheduling behavior of all outpatient physicians, they do reflect the actual experiences of patients who rely on online booking platforms to access care. Given the growing use of digital scheduling systems, these platforms increasingly shape how patients search for and obtain appointments. Consequently, the observed patterns may become more relevant for patient access to care over time.

Second, the platform’s proprietary ranking algorithms are opaque; while we see a correlation suggesting an “earliest‐appointment” prioritization, other factors may influence the ranking, given that this association does not imply causation.

Third, our audit design captures only the first available slots and cannot account for cancellations, patient no‐shows, or subsequent appointment opportunities. The “first available appointment” is a useful and reproducible audit metric for comparing what the platform exposes to different insurance profiles at a given moment. However, it is only an approximation of actual access. Real patient pathways may differ because patients can accept cancellations, request urgent slots, choose a preferred physician, switch providers, or obtain appointments through nondigital channels. As a result, the observed waiting-time gap may not translate one-to-one into realized delays in care, especially if SHI and PHI patients differ in their ability or willingness to use such alternative pathways. In particular, because the study measures visible first available slots rather than completed appointments, the estimated differences should be interpreted as differences in booking opportunity, not necessarily as direct differences in treatment receipt.

Fourth, 2 profiles were not perfectly identical because they were based on real first-time user accounts that differed in gender and age; however, the age difference of 6 years can be considered negligible. In addition, we conducted a robustness check by switching SHI and PHI status across the profiles and reassessed the first 20 listings. This analysis confirmed the observed differences by insurance status, suggesting that the gap between SHI and PHI patients is unlikely to be driven by gender or age. The validation, therefore, supports the robustness of the insurance gradient, but it does not perfectly rule out gender- or age-based confounding.

Fifth, distributions of waiting times were skewed, so while the mean reflects the average experience of the patient profile in this study, the findings are likely to differ for all patients. This is reflected in the outlier exclusion, where cardiology was no longer significant and was mainly driven by the outliers.

Finally, although specialty-level estimates were directionally consistent with the main finding, these subgroup analyses were exploratory and underpowered in several strata. Accordingly, apparent between-specialty differences should not be interpreted as definitive evidence of specialty-specific effects. The Bonferroni-adjusted results reduce concerns about multiple testing but do not eliminate the power issues.

### Conclusions

Online scheduling platforms, while improving convenience, appear to reaffirm offline inequities in specialist access: statutory‐insured patients wait substantially longer than their PHI counterparts. These disparities are consistent across multiple specialties and robust to validation analyses. Importantly, the observed inequality reflects the structural features of Germany’s dual insurance system rather than being created by the platform itself. However, if platform rankings are influenced by waiting times, differences in waiting times between SHI and PHI patients could further amplify existing inequalities by making practices with earlier PHI than SHI availability more prominent on the platform. As online booking becomes an increasingly dominant mode of access to outpatient care, systematic monitoring of its distributional effects is essential. Policymakers and platform operators should consider regulatory and design interventions that promote equitable access and greater transparency in the factors influencing platform rankings, helping to ensure that digitalization contributes to fairness rather than reinforcing existing inequalities in health care access.

## Supplementary material

10.2196/82452 Multimedia Appendix 1 Variable list.

10.2196/82452 Multimedia Appendix 2 Estimated Kassenärztliche Vereinigung market share of Doctolib in Berlin based on practice specialization and registry data.

10.2196/82452 Checklist 1 STROBE checklist.

## Abbreviations

ENT: ear, nose, and throat
KV: Kassenärztliche Vereinigung (Association of Statutory Health Insurance Physicians)
PHI: private health insurance
SHI: statutory health insurance
STROBE: Strengthening the Reporting of Observational Studies in Epidemiology

## References

[1] Angerer S, Waibel C, Stummer H (2019). Discrimination in health care: a field experiment on the impact of patients’ socioeconomic status on access to care. Am J Health Econ.

[2] Kim CY, Wiznia DH, Roth AS, Walls RJ, Pelker RR (2016). Survey of patient insurance status on access to specialty foot and ankle care under the Affordable Care Act. Foot Ankle Int.

[3] Rhodes KV, Kenney GM, Friedman AB (2014). Primary care access for new patients on the eve of health care reform. JAMA Intern Med.

[4] von dem Knesebeck O, Klein J (2025). Attitudes towards the dual health insurance system and inequalities in health care in Germany—results of a population survey. BMC Health Serv Res.

[5] Breitenbach A, Heinrich M (2023). Diskriminierung im deutschen Krankenversicherungssystem: Werden gesetzlich Versicherte bei der Terminvergabe von Fachärzten benachteiligt? [Report in German]. https://www.ssoar.info/ssoar/bitstream/handle/document/85085/ssoar-2023-breitenbach_et_al-Diskriminierung_im_deutschen_Krankenversicherungssystem_Werden.pdf?sequence=1&isAllowed=y&lnkname=ssoar-2023-breitenbach_et_al-Diskriminierung_im_deutschen_Krankenversicherungssystem_Werden.pdf.

[6] Heinrich N, Wübker A, Wuckel C (2018). Waiting times for outpatient treatment in Germany: new experimental evidence from primary data. Jahrb Natl Ökon Stat.

[7] Roll K, Stargardt T, Schreyögg J (2012). Effect of type of insurance and income on waiting time for outpatient care. Geneva Pap Risk Insur Issues Pract.

[8] Muschol J, Gissel C (2021). COVID-19 pandemic and waiting times in outpatient specialist care in Germany: an empirical analysis. BMC Health Serv Res.

[9] Werbeck A, Wübker A, Ziebarth NR (2021). Cream skimming by health care providers and inequality in health care access: evidence from a randomized field experiment. J Econ Behav Organ.

[10] Lungen M, Stollenwerk B, Messner P, Lauterbach KW, Gerber A (2008). Waiting times for elective treatments according to insurance status: a randomized empirical study in Germany. Int J Equity Health.

[11] Luque Ramos A, Hoffmann F, Spreckelsen O (2018). Waiting times in primary care depending on insurance scheme in Germany. BMC Health Serv Res.

[12] Atherton H, Eccles A, Poltawski L, Dale J, Campbell J, Abel G (2024). Investigating patient use and experience of online appointment booking in primary care: mixed methods study. J Med Internet Res.

[13] Zhao P, Yoo I, Lavoie J, Lavoie BJ, Simoes E (2017). Web-based medical appointment systems: a systematic review. J Med Internet Res.

[14] Nabila N, Ayuningtyas D (2024). The effectivity of outpatient waiting time in hospital through online or web-based reservation (literature review). Asian J Eng Soc Health.

[15] Kurtzman GW, Keshav MA, Satish NP, Patel MS (2018). Scheduling primary care appointments online: differences in availability based on health insurance. Healthc (Amst).

[16] Samorani M, Harris S, Blount LG, Lu H, Santoro MA (2021). Overbooked and overlooked: machine learning and racial bias in medical appointment scheduling. Manuf Serv Oper Manag.

[17] von Elm E, Altman DG, Egger M (2008). The Strengthening the Reporting of Observational Studies in Epidemiology (STROBE) statement: guidelines for reporting observational studies. J Clin Epidemiol.

[18] (2005). Directive 2005/36/EC of the European Parliament and of the Council of 7 September 2005 on the Recognition of Professional Qualifications (text with EEA relevance). European Union.

[19] Bonferroni CE (1935). Il calcolo delle assicurazioni su gruppi di teste [Article in Italian]. Studi in onore del professore salvatore ortu carboni.

[20] (2023). FAQ: humanities and social sciences. Deutsche Forschungsgemeinschaft.

