# Supplementary material for: How Online Scheduling Platforms Affect Insurance-Based Disparities in Access to Specialist Outpatient Care in Berlin, Germany: Cross-Sectional Audit Study
Source: J Med Internet Res. 2026 Jun 15;28:e82452. doi: 10.2196/82452 (PMC13268634; doi:10.2196/82452)
Supplement: Multimedia Appendix 1 [file jmir-v28-e82452-s001.docx]

**Table S2.** Overview of extracted variables from the platform and their definitions

| Variable | Definition | Connection |
| --- | --- | --- |
| N. of listing | Unique identifier as serial number | Each listing has one number  One practice might have several listings |
| Speciality | General Surgery, Ophthalmology, Obstetrics & Gynecology, Vascular Surgery, Dermatology, ENT, Cardiology, Pediatrics, Neurology, Orthopedics, Radiology, Urology | Each listing is attributed to exactly one specialty |
| Practice name and (partly) address | Copied identifier how it was used within Doctolib  e.g.: Mrs. Test Test, Gynecologist, Kurfürstendamm 150, 10707 Berlin | All listings include the practice name, three specialties additionally provided the address |
| Bookable for SHI | Yes (1) or No (0) according to whether the SHI Profile had the option to book an appointment | For each practice listing availability of a SHI appointment was documented |
| Bookable for PHI | Yes (1) or No (0) according to whether the PHI Profile had the option to book an appointment | For each practice listing availability of a PHI appointment was documented |
| First available appointment SHI | Appointment date that is closest to assessment date | For each practice (9) if none available as indicator for missing value |
| First available appointment SHI | Appointment date that is closest to assessment date | For each practice (9) if none available as indicator for missing value |
| Reason not bookable SHI | If available reason why the profile could not book an appointment (details Fig.1) | e.g. only special appointments available, or self-pay options |
| Reason not bookable PHI | If available reason why the profile could not book an appointment (details Fig.1) | e.g. no appointments available over Doctolib at all, at this moment no further appointment slots released by the practice |
| Day of data collection/assessment | Exact date (tt.mm.yyy) |  |
| Weekday of data collection | Weekday extracted from assessment date (Monday, Tuesday… |  |
| Waiting time for SHI profile | Days between assessment date and appointment date | For each practice |
| Waiting time for PHI profile | Days between assessment date and appointment date | For each practice |
| Difference in waiting times | Waiting time for SHI profile - Waiting time for PHI profile |  |
| Rank on Doctolib | Rank on doctolib. (e.g: Rank 1: First listing on the first page; Rank 20 last listing on the first page; Rank 21 first listing on the second page; Rank 40 last listing on the second page…) | Retrospectively assessed using practices with address and screenshots of pages from the assessment day to determine the exact place of the listing on the day of assessment. |
