# Supplementary material for: How Online Scheduling Platforms Affect Insurance-Based Disparities in Access to Specialist Outpatient Care in Berlin, Germany: Cross-Sectional Audit Study
Source: J Med Internet Res. 2026 Jun 15;28:e82452. doi: 10.2196/82452 (PMC13268634; doi:10.2196/82452)
Supplement: Multimedia Appendix 2 [file jmir-v28-e82452-s002.docx]

**Table S3.** Estimated market share of Doctolib in Berlin based on practice specialization and registry data

| **KV Group** | **Registered in KV (n)** | **SHI Bookable (n)** | **PHI Bookable (n)** | **Bookable Practices (n)** | **% SHI Bookable of KV** | **% Evaluable Cases*** |
| --- | --- | --- | --- | --- | --- | --- |
| Surgery and Orthopedics** | 689 | 189 | 287 | 175 | 27,4% | 25,4% |
| Ophthalmology | 349 | 20 | 57 | 20 | 5,7% | 5,7% |
| Obstetrics and Gynecology | 730 | 108 | 209 | 103 | 14,8% | 14,1% |
| Dermatology and Venereology | 228 | 18 | 172 | 18 | 7,9% | 7,9% |
| Ear, Nose and Throat (ENT) / Phoniatrics and Ped. audiology | 281 | 82 | 116 | 75 | 29,2% | 26,7% |
| Internal Medicine with Specialty Practice | 587 | 15 | 87 | 14 | 2,6% | 2,4% |
| Pediatrics | 410 | 12 | 22 | 12 | 2,9% | 2,9% |
| Neurology / Psychiatry | 405 | 2 | 32 | 2 | 0,5% | 0,5% |
| Radiology | 261 | 38 | 53 | 38 | 14,6% | 14,6% |
| Urology | 183 | 36 | 103 | 35 | 19,7% | 19,1% |
| Total | 4123 | 520 | 1138 | 492 | 12,6% | 11,9% |
| * Evaluable Cases = Bookable Practices for both groups  **This group combines the single specialties general surgery, vascular surgery and orthopedics. | | | | | | |
