# Supplementary material for: How Online Scheduling Platforms Affect Insurance-Based Disparities in Access to Specialist Outpatient Care in Berlin, Germany: Cross-Sectional Audit Study
Source: J Med Internet Res. 2026 Jun 15;28:e82452. doi: 10.2196/82452 (PMC13268634; doi:10.2196/82452)
Supplement: Checklist 1 [file jmir-v28-e82452-s003.pdf]

**Table S1.** Strobe Checklist

| Section | Item | STROBE Recommendation | Response |
|---|---|---|---|
| Title & Abstract | 1a | Indicate study design | "Cross-Sectional Observational Study" in title |
| | 1b | Informative and balanced summary | Abstract includes design, methods, results, and conclusions |
| Introduction | 2 | Background/rationale | Explains disparity in insurance-based access and relevance of online platforms |
| | 3 | Objectives | Primary and two secondary clearly defined |
| Methods | 4 | Study design | Cross-sectional audit via simulated online booking |
| | 5 | Setting | Berlin, Germany; Jan 6 – Feb 26, 2025 |
| | 6 | Participants | Inclusion and exclusion based on booking availability and insurance types; specialists preselected via Frankfurt pre-study |
| | 7 | Variables | Insurance type, wait time, specialty, booking constraints |
| | 8 | Data sources/measurement | Manual simulated booking with saved pages; same-day SHI/PHI data |
| | 9 | Bias | Order of appearance from platform respected; static snapshot of results saved to reduce bias |
| | 10 | Study size | 1867 screened, 492 included; flow diagram available |
| | 11 | Quantitative variables | Wait time (in days); stratified by specialty and practice |
| | 12a | Statistical methods | Descriptive stats; paired t-tests; specialty-stratified analysis |
| | 12b | Subgroups | By medical specialty |
| | 12c | Missing data | Missing appointment types were exclusion criteria; documented |
| | 12d | Sensitivity analysis | Top 5% of longest wait times excluded as sensitivity analysis; results tested for robustness |
| Results | 13a | Numbers at each stage | Flow diagram showing inclusion/exclusion |
| | 14a | Descriptive data | Practice characteristics, booking constraints, specialties |
| | 15 | Outcome data | SHI vs. PHI wait times at each provider |
| | 16 | Main results | Difference in mean/median wait times; algorithm influence |
| | 17 | Other analyses | Fair-access deprioritization & self-pay offers to SHI patients |
| Discussion | 18 | Key results | PHI patients favored; online systems replicate offline disparities |
| | 19 | Limitations | Platform sample, algorithm opacity, no phone-based comparison |

| | | | |
|---|---|---|---|
| | 20 | Interpretation | Results interpreted in context of German health system and digital transformation |
| | 21 | Generalizability | Applies to urban Germany; may differ in rural areas or non-digital bookings |
| Other Info | 22 | Funding | No funding received |
| | Ethical | No ethical vote | No personal data collected, no participants, only publicly available data was observed; no bookings were made; no ethical consent was applied for. |
